# Improved Resting-State Functional MRI Using Multi-Echo Echo-Planar Imaging on a Compact 3T MRI Scanner with High-Performance Gradients

**DOI:** 10.3390/s23094329

**Published:** 2023-04-27

**Authors:** Daehun Kang, Myung-Ho In, Hang Joon Jo, Maria A. Halverson, Nolan K. Meyer, Zaki Ahmed, Erin M. Gray, Radhika Madhavan, Thomas K. Foo, Brice Fernandez, David F. Black, Kirk M. Welker, Joshua D. Trzasko, John Huston, Matt A. Bernstein, Yunhong Shu

**Affiliations:** 1 Department of Radiology, Mayo Clinic, Rochester, MN 55905, USA; kang.daehun@mayo.edu (D.K.);; 2 Department of Physiology, Hanyang University, Seoul 04763, Republic of Korea; 3 Mayo Clinic Graduate School of Biomedical Sciences, Mayo Clinic, Rochester, MN 55905, USA; 4 GE Global Research, Niskayuna, NY 12309, USA; 5 GE Healthcare, 78530 Buc, France

**Keywords:** fMRI, BOLD, resting state, multi-echo EPI, compact 3T, gradient system, slew rate

## Abstract

In blood-oxygen-level-dependent (BOLD)-based resting-state functional (RS-fMRI) studies, usage of multi-echo echo-planar-imaging (ME-EPI) is limited due to unacceptable late echo times when high spatial resolution is used. Equipped with high-performance gradients, the compact 3T MRI system (C3T) enables a three-echo whole-brain ME-EPI protocol with smaller than 2.5 mm isotropic voxel and shorter than 1 s repetition time, as required in landmark fMRI studies. The performance of the ME-EPI was comprehensively evaluated with signal variance reduction and region-of-interest-, seed- and independent-component-analysis-based functional connectivity analyses and compared with a counterpart of single-echo EPI with the shortest TR possible. Through the multi-echo combination, the thermal noise level is reduced. Functional connectivity, as well as signal intensity, are recovered in the medial orbital sulcus and anterior transverse collateral sulcus in ME-EPI. It is demonstrated that ME-EPI provides superior sensitivity and accuracy for detecting functional connectivity and/or brain networks in comparison with single-echo EPI. In conclusion, the high-performance gradient enabled high-spatial-temporal resolution ME-EPI would be the method of choice for RS-fMRI study on the C3T.

## 1. Introduction

To study brain activity based on hemodynamic signals, blood-oxygen-level-dependent (BOLD) contrast has been widely used in functional MRI (fMRI) [1,2]. The gradient-recalled-echo echo-planar imaging (GRE-EPI) pulse sequence has been a workhorse for localizing brain activation regions and/or assessing intrinsic resting-state (RS) functional connectivity (FC) due to its sensitivity to BOLD signal. It has a short sampling time of a few seconds or less for whole-brain imaging. Since the source of the BOLD signal is caused by variation in magnetic susceptibility driven by vascular flow and relative deoxyhemoglobin concentration, T_2_^*^ change is directly related to BOLD signal [3]. For a conventional single-echo/one-echo (OE) GRE-EPI acquisitions, the echo time (TE) used at 3T is typically set to approximately 30 ms, which is lower than the optimum (at TE~T_2_^*^) and represents a good compromise between BOLD sensitivity, image quality, and temporal resolution without excessive signal losses [4,5,6]. In landmark functional MRI (fMRI) studies, such as the Human Connectome Project (HCP), Alzheimer Disease Neuroimaging Initiative (ADNI) and Adolescent Brain Cognitive Development (ABCD) studies, all recommend the imaging protocol of spatial and temporal resolutions less than 2.5 mm isotropic voxel and 1 s repetition time (TR), respectively [7,8,9], which could be achieved with advanced imaging acceleration techniques, such as parallel imaging and simultaneous-multi-slice (multi-band, MB) imaging [10,11,12,13]. Although studies with multi-subject averaging with large sample sizes have shown group-level trends in RSFC, striving for better image SNR or temporal SNR (TSNR) remains a continuing effort in order to improve measurement robustness for individual subject [14,15].

Compared to single-echo acquisition, multi-echo (ME) is a fascinating approach that can address various goals in one acquisition, including sensitivity/SNR improvement and quantification of T_2_^*^ relaxation time for clinical and basic neuroscience research. The ME scheme has been demonstrated with robust performance in various applications, including in a line-scanning fMRI with high spatiotemporal resolutions [16], in a combined gradient- and spin-echo acquisition for simultaneous quantify T_2_^*^ and T_2_ relaxation time quantification [17], and in blood flow measurements to quantify T_2_^*^ relaxation time [18]. Recently, a deep learning model was proposed for end-to-end parametric mapping for an overlapped multi-echo sequence [19]. Although a single echo train was used, overlapping multi-echo signals can be generated with multiple excitations. ME fMRI has been increasingly utilized in RS-fMRI studies as it can yield additional TE dependence information. For example, a study used a convolutional neural network to extract a BOLD pulsatility map from ME RS data [20]. ME fMRI combined with independent component analysis (ICA)-based artifact reduction technique has been applied to achieve improved BOLD signal quality in high-motion cohort groups like children [21]. As a promising technological advancement in this regard, ME fMRI has been proposed and shown to be advantageous for better denoising and boosting the reliability of functional connectivity [22,23,24,25].

ME acquisition scheme generally requires repetitive echo train measurements, which can compromise temporal resolution and lead to undesirable late echo time to go past the T_2_^*^ decay window. One solution to this is to use a high gradient slew rate and reduce the duration of the EPI echo spacing. Advancements in MRI scanner hardware have enabled high-performance gradients, which can directly impact the implementation and performance of the ME acquisition. In this paper, we will use a compact 3T (C3T) scanner as a technology demonstrator for ME-fMRI. A lightweight, low-cryogen C3T MRI system is equipped with a high-performance gradient system mainly for brain imaging. The gradient coil system on the C3T has an inner diameter of 42 cm, yielding a 26 cm diameter of spherical volume for whole-brain imaging. Due to its small size, it can operate at a peak slew rate of 700 T/m/s with a peak amplitude of 80 mT/m on all gradient axes simultaneously [26,27,28,29,30] without peripheral nerve stimulation limitations [31,32]. In comparison, conventional whole-body MR scanners typically offer up to 200 T/m/s gradient slew rate and 40–80 mT/m peak gradient amplitude. The high-performance gradient system of the C3T is particularly beneficial for EPI-based acquisition. The higher gradient slew rate effectively minimizes the echo spacing and thereby reduces the readout duration [33]. Specifically, with the intended spatial-temporal resolution, the echo spacing on the C3T is 17.9 ms, which has been reduced by 43% from the echo spacing of 31.2 ms on a whole-body scanner with a 200 T/m/s slew rate. The third echo time is 47.8 ms, which can yield a late echo with substantial signal and T_2_^*^ information. On the contrary, the whole-body scanner could only obtain a third echo at 81.5 ms, with the echo signal too weak to be useful. Additional benefits of reduced echo spacing includes greatly improved magnetic-susceptibility-induced geometric distortion [33,34,35]. Additionally, the shortened readout improves SNR obliviating the need for a partial Fourier sampling [36]. Furthermore, the shortened readout on the C3T enables minimal TR increase, which is helpful when high temporal resolution is desired.

Although ME acquisition has been well demonstrated to be superior for fMRI compared to OE acquisition [4,25,37,38,39], previous studies using ME acquisitions were conducted on conventional whole-body MRI systems with imaging parameters that marginally met the requirements for fMRI, such as a ~2 to 3 s TR and/or greater than 3 mm isotropic resolution with partial Fourier acquisition to cover the whole brain. fMRI studies prioritizing higher spatial or temporal resolutions while still maintaining whole brain coverage could not choose ME fMRI despite its benefits [40]. Advanced gradient performance can help break the tradeoffs by enabling the desired spatial and temporal resolutions for ME fMRI that are similar to those of OE fMRI used in landmark fMRI studies [7,8,9].

The purpose of this study was to perform a comprehensive investigation on the feasibility and utility of ME fMRI imaging with a high spatiotemporal resolution by comparing it with an OE fMRI acquisition with matched spatial resolution on the C3T. Our hypothesis is that ME fMRI has improved RS FC detectability on the C3T when compared with OE fMRI, the spatiotemporal resolution of which is similar to those used in the landmark fMRI studies. For a fixed acquisition time, the counterpart OE fMRI has more time frames compared to the ME fMRI. It is not clear if the advantage of ME fMRI still holds, considering the loss of temporal resolution at the spatial resolution. Thus, data from both ME and OE acquisitions were obtained on the C3T from healthy volunteers with the same acquisition parameters, except that OE uses a shorter TR. The performance of ME to OE acquisition was assessed by evaluating explained variance, SNR and TSNRs. The signal intensity recovery by ME acquisition was investigated using cortical coverage analysis and ROI-based functional connectivity. Additionally, seed- and independent-component-analysis (ICA)-based analyses in functional connectivity were used to determine the effectiveness of ME fMRI. It was demonstrated that ME-EPI provides superior sensitivity and accuracy for detecting functional connectivity and/or brain networks in comparison with OE-EPI. 

## 2. Materials and Methods

### 2.1. Data Acquisition

Following a study protocol approved by the Institutional Review Board, 24 healthy subjects (age = 35.0 ± 10.7 years; 18 females/6 males) participated in this study after written informed consent was obtained. All subjects were scanned on the C3T using a 32-channel brain coil (Nova Medical, Inc., Wilmington, MA, USA). During the RS-fMRI scans, all subjects were instructed to remain still and fix their gaze on the center of a mirror mounted on the head coil. Independent measures of physiological variables (cardiac and respiration) were recorded.

RS-fMRI experiments were conducted using a ME MB gradient-echo EPI sequence with blipped-controlled aliasing in parallel imaging technique [10,37]. The fMRI datasets were separately acquired for OE and ME (three echoes) acquisitions. A whole-brain fMRI protocol, including the following imaging parameters were applied in Table 1. ME acquisition was consecutively repeated twice to fill 10 min of scan duration due to a scanner system memory software limit on the total number of reconstructed images per series (GE Healthcare, DV26) rather than hardware restriction.

As an anatomical reference, a high-resolution T_1_-weighted image (magnetization-prepared rapid acquisition with gradient echo, or MPRAGE) was obtained on the C3T with the following parameters: TR= 5.4 ms, TE = 2.4 ms, inversion time = 1000 ms, FA = 8°, resolution = 1.0 × 1.0 × 1.0 mm^3^, field of view = 25.6 × 16.6 × 25.6 cm^3^. Gradient nonlinearity (GNL) induced distortion on all fMRI and anatomical images obtained with the C3T was corrected in the image domain retrospectively using a tenth-order inline GNL correction (gradwarp) algorithm [30,31].

### 2.2. Data Preprocessing for fMRI Datasets

The preprocessing procedures for RS-EPI datasets were all carried out using AFNI (Analysis of Functional NeuroImages, https://afni.nimh.nih.gov/, accessed on 26 March 2021)’s suite of programs [41]. For preprocessing and artifact reduction of the RS-EPI data, the following steps were performed in sequence: truncation by initial ten volumes to yield steady-state magnetization, de-spiking (‘3dDespike’ in AFNI), physiological noise elimination including cardiac and respiratory artifacts (‘RETROICOR’ and ‘RVT’) [42,43], slice acquisition timing correction (‘3dTshift’ in AFNI), time-series alignment, Legendre polynomial detrending and motion- and hardware-related linear regressions [44,45,46], in order. For the ME dataset, a T_2_^*^-weighted echo combination was performed between the time-series alignment and the detrending and the regressions [4,38,47] by using AFNI (‘@compute_OC_weights’). All datasets were evaluated for artifacts due to abrupt head motion, passing the sudden motion detection of AFNI at the threshold level of 0.2 mm for the Euclidean L2 norm of motion displacement during each TR interval [45].

For functional connectivity (FC) estimation, the artifact-reduced residual time series were bandpass-filtered with a frequency range of 0.009 to 0.1 Hz and smoothed by a Gaussian kernel with a 4 mm full-width-at-half-maximum on volume dataset (‘3dTproject’ in AFNI). The preprocessed datasets were spatially normalized into the 2.0-mm-isotropic-resolution MNI template of ‘MNI152_T1_2009c+tlrc.HEAD’ by ‘auto_tlrc’ in AFNI. For FC investigation, the two ME datasets were concatenated after time-series z-score normalization as performed in the previous study [48] to render the final dataset with ~10-min length.

### 2.3. Data Analysis

#### 2.3.1. Explained Variance Evaluation

To examine the change in signal variance using a model component, namely a regressor or echo combination, the marginal *R*^2^ value was introduced to represent the proportion of the signal variance observed without individual preprocessing steps relative to the variance observed with all preprocessing steps, as performed in the previous study [44]. The value of *R*^2^ was evaluated from:

(1) $$R_{step}^{2}\equiv 1-\frac{{SS}_{total}}{{SS}_{step}},$$

where *SS_total_* was the error sum of squares with all preprocessing steps, and *SS_step_* was the error sum of squares with all preprocessing steps except for a certain step. Without bandpass filtering and spatial smoothing, *R*^2^ were evaluated for the conventional artifact-related regressors of the RETROICOR [42], ANATICOR [44], motion and global cerebrospinal fluid (CSF) signal, and for the T_2_^*^-weighted echo combination only in ME datasets. For comparison between OE and ME acquisitions, ME datasets before the time-course scaling were used and the mean *R*^2^ values were calculated in gray matter (GM) and white matter (WM) voxels across all subjects.

To evaluate the dependence between artifact/noise removed by regressors and the T_2_^*^-weighted echo combination, the Pearson correlation coefficients were calculated. The numbers of regressors for RETROICOR [42] (up to 2nd order, including 5 regressors of RVT [43]), motion (six parameters for rigid-body motion and their first order derivative), and global-CSF artifacts (first 3 principal components of lateral ventricles) were thirteen, twelve and three for each dataset, respectively. The single time series for each voxel was collected as an ANATICOR regressor. The noise removed by the echo combination was estimated by subtraction between echo-combined and mid-echo-only time courses. The correlation coefficients calculated across regressions and subjects were averaged by mean root square for the summary. Additionally, SNR and TSNR of ME and OE datasets were investigated across GM and WM voxels [49], where TSNR were derived with/without the regressions to compare artifact reduction effect on TSNR in ME and OE datasets.

#### 2.3.2. ROI-Based FC Analysis

The effect of echo combination on signal intensity recovery was investigated for OE and ME datasets. A mask was created with the base EPI image of each dataset binarized by ‘3dAutomask’ in AFNI and converted onto a 3D surface model of ‘suma_TT_N27′ provided in SUMA [50]. The cortical coverage was visualized by overlapping each EPI mask with the gray matter on the corresponding anatomy image [35]. The cortical coverage ratio was evaluated as the percentage of overlap between the averaged EPI masks across subjects and the gray matter on the surface model and [35].

The effect of signal intensity enhancement of echo combination on FC was also investigated by ROI-based FC. 168 cortical parcellations for an individual anatomical image were defined as ROIs by FreeSurfer software [51,52]. The inter-ROI FC was evaluated as the Pearson correlation coefficient between the time courses of the two ROIs. Fisher-transformed FCs composed an FC matrix of 168 × 168. Due to regional signal dropout in images, which depended on the subject’s anatomical shape and position against the B0 field, a paired Student’s *t*-test for group comparison was applied to the FC matrices obtained from OE and ME datasets from 18 subjects. Inter-ROI FCs were thresholder with a statistically significant difference (uncorrected *p* < 0.01) and a meaningful FC value (Fisher(r)) > 0.3) in either OE or ME FCs, and the numbers of the inter-ROI FCs survived by the two thresholds were counted.

#### 2.3.3. Seed-Based FC Map

To examine seed-based FC extents, 10 ROIs adopted from AAL3v1 [53] were selected as the seeds for the spatially normalized preprocessed datasets, including anterior cingulate cortex (ACC), insula (Ins), hippocampus (Hipp), posterior cingulate cortex (PCC), and precentral gyrus (PCG) on both hemispheres [54,55]. To evaluate an individual FC map, the Pearson correlation coefficients were calculated between the time courses of each voxel and the seed regions, which were converted into a z-score by Fisher transformation. Average FC maps for both OE and ME datasets were calculated by unpaired two-sample Student’s t-tests, including one-sample tests by ‘3dttest++’ in AFNI with a covariate of global correlation (GCOR) [56] and corrected by the FDR algorithm (‘3dFDR’ in AFNI). The group-level FC maps were statistically thresholded by the FDR-corrected *p*-value < 0.001. After statistical thresholding, the FC-magnitude threshold was applied to the seed-based group-level FC maps, and the number of voxels that survived was evaluated as the extent of FC.

#### 2.3.4. ICA-Based FC Detection

Independent component analysis (ICA)-based functional connectivity was investigated by using concat-ICA and dual regression methods. Concat-ICA was used to identify a set of independent component (IC) maps from the whole group of OE and ME datasets, which was performed by using the ‘melodic’ command in FSL with an option of 20 dimensions [57,58,59]. Among 20 IC maps, 8 IC maps were identified as brain networks, including default mode network (DMN), lateral visual areas (LOcc), medial visual areas (MVis), right and left frontal-parietal networks (RFPN and LFPN), auditory network (Aud), executive control network (ECN), and sensorimotor network (Sens) [57]. The whole-group IC maps were used as the inputs for dual regression and as the group truth with a statistical threshold to assess OE/ME group results derived by dual regression.

Dual regression was conducted by ‘dual_regression’ provided in FSL with the following two steps [58,59,60]. The first step was to identify a subject-specific time course using spatial regression of the group IC maps derived by concat-ICA. The second step was to identify a subject-specific spatial map using temporal regression of the subject-specific time course. To investigate the scan time dependence, i.e., the effect of scan time length on the sensitivity and specificity of ICA-derived brain network, each subject-specific dataset was truncated to time lengths ranging from 2 to 9 min at increments of 1 min. The same dual regression was applied to each dataset with different time lengths as the group IC maps. For a statistical comparison between the ME and the OE datasets, the OE/ME groups of individual spatial IC maps were tested by using ‘randomise’ command of the FSL’s randomise permutation-testing tool (N = 5000). 

In this study, whole-group IC maps were assumed to be the ground truth for brain networks [57,60]. OE-/ME-group IC maps derived from OE/ME datasets were assessed in terms of sensitivity, specificity, and accuracy. For the quantitative assessment, the OE-/ME-group IC maps were binarized by a threshold of *p* < 0.001 to create the corresponding masks. The numbers of voxels were counted as true positives (TP), true negative (TN), false positive (FP), and false negative (FN) based on the OE-/ME-group IC map mask on the corresponding whole-group IC map mask. Sensitivity, 1—specificity (as false positive rate, FPR), and accuracy for each IC map were evaluated as followings, respectively.

(2) $$Sensitivity=\frac{TP}{TP+FN}$$

(3) $$False\;positive\;rate=1-\frac{TN}{TN+FP}$$

(4) $$Accuracy=\frac{TP+TN}{TP+TN+FP+FN}$$

## 3. Results

### 3.1. Data Selection for Analysis

Among the total of 24 volunteers, two subjects had 70 and 48 time frames censored by the criterion in concatenated ME data and 22 and 32 in OE data, respectively, which were quite larger than the number of censored time frames of other subjects. The datasets from the subjects were considered to be contaminated with high motion [61] and excluded for subsequent analysis. Four volunteers were scanned only with OE fMRI, without ME fMRI. Thus, 22 sets of 11-min for OE datasets and 36 sets of 5-min for ME datasets (2 sets from 18 individual subjects) were used for further analysis. 

The average translational motion per TR across datasets were 0.028 ± 0.010 mm and 0.027 ± 0.010 mm for OE and ME acquisitions, respectively, which corresponded to 0.033 ± 0.012 mm and 0.033 ± 0.012 mm average motion per second, which was much smaller than the censoring criterion of 0.2 mm. Time frames with motion exceeding 0.2 mm were censored in each dataset. 1.8 (±2.7) and 1.4 (±2.4) of time frames were censored on average (±standard deviation) for OE and ME acquisitions, respectively. Thus, the average number of samples used was 949 ± 3 and 309 ± 2 for OE and ME session, respectively. After concatenation of ME datasets, all preprocessed datasets ended up with various data length ranging from 10 min 58 s and 9 min 26 s and they were all truncated to 9-min length for comparison purpose, where 772 and 575 time frames were used for further FC analysis of OE and ME acquisitions, respectively.

### 3.2. Explained Variance

**Figure 1:**
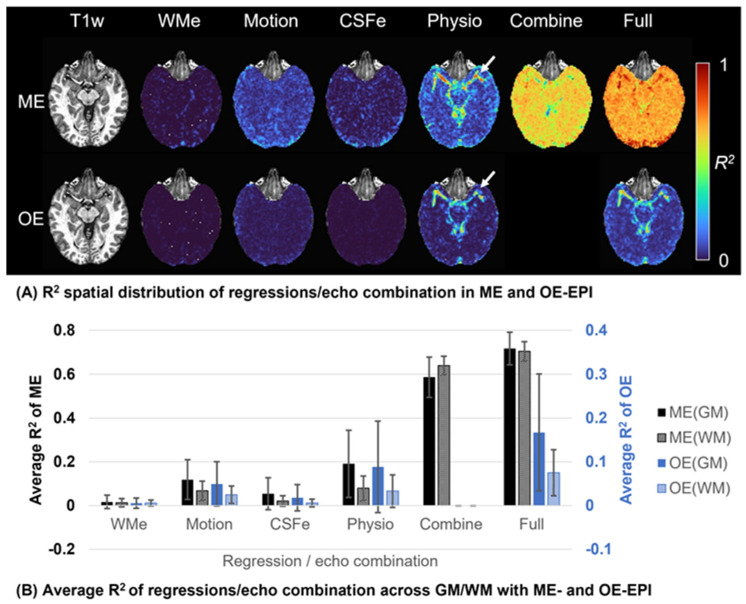

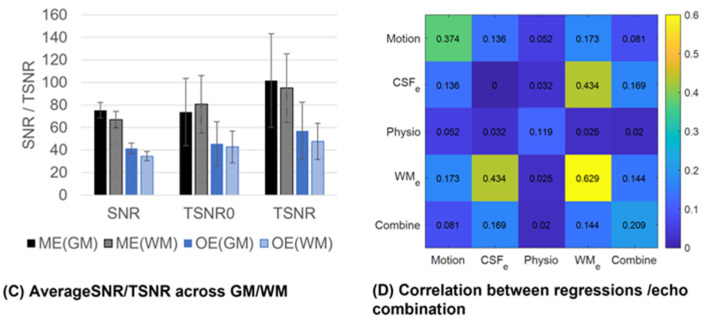
(**A**) The explained variance (*R*^2^) maps for each regressor or echo combination of a single subject with ME and OE datasets. The *R*^2^ maps are derived by regressions related with eroded local white matter (WMe), six rigid-body motion parameter estimates (Motion), eroded four large ventricles (CSFe), and recorded respiration and cardiac pulsation (Physio) and by T_2_^*^-weighted echo combination (Combine). The total effect on *R*^2^ map by all regressions and echo combination is given (Full). The T_1_-weighted anatomical images with the corresponding locations are also shown at left. (**B**) Average *R*^2^ of each regressor or echo combination were plotted for ME and OE datasets across gray and white matters of subjects. Note that average *R*^2^ of OE datasets has a different scale on the right side of the plot. Additionally, (**C**) SNR and TSNRs of ME and OE datasets are present. TSNR0 denotes TNSR evaluated without regression. (**D**) Root mean square of correlation coefficients between regressors and noises removed by echo combination to assess the dependence between time series of artifacts/noises removed. Through plots (**B**,**C**), error bars denote global means and standard deviations for voxels of gray and white matter across the corresponding datasets.

Figure 1A showed an example of *R*^2^ spatial maps in ME and OE acquisitions. A great reduction in signal variance by echo combination can be globally visualized (‘Combine’ in Figure 1A), except for regions near large vessels where *R*^2^ for the RETROICOR regressors dominates as indicated in ‘Physio’ of Figure 1A. In Figure 1B, average *R*^2^ in the GM and WM were both evaluated for conventional regressors and T_2_^*^-weighted echo combination of ME acquisitions and for the regressors of OE acquisitions as reference. The conventional regressors for ME acquisitions showed a similar trend in *R*^2^ values with those for OE acquisitions except for the scale. *R*^2^ for echo combination were substantially higher than *R*^2^ for other regressors. It showed that a fairly large portion of signal variance was removed through echo combination. For echo combination, *R*^2^ in WM tended to be slightly larger than in GM. 

The reduced signal variance led to improve SNR and TSNR of ME acquisitions in Figure 1C. Note that TSNR0 and TSNR were evaluated without and with artifact regression processing, respectively. In terms of TSNR, the artifact regression was more effective with ME datasets than OE datasets. TSNR gains in ME verse OE acquisitions were approximately 1.8 and 2.0 times on GM and WM, respectively. The OE acquisition has the number of time frames 34% more than the ME fMRI (700 vs. 940 ms). Considering TSNRs with the numbers of time frames like $TSNR\cdot \sqrt{\#\;of\;time\;frames}$ [13], its gains in ME verse OE acquisitions were approximately 1.56 and 1.74 times on GM and WM in the same scan time, respectively. In terms of $TSNR\cdot \sqrt{\#\;of\;time\;frames}$ on GM, approximately 2.4 times longer scan time for OE acquisition would be needed to have an equivalent value to ME acquisition.

Correlations of noise reduced by echo combination were calculated and compared to those among other regressors in Figure 1D. The noise removed by echo combination was relatively independent from other regressors or itself. While a voxel-wise regressor of ANATICOR [44] tended to be highly correlated to regressors of adjacent voxels, the voxel-wise noise removed by echo combination showed the low-level correlation with noises removed in adjacent voxels.

### 3.3. ROI-Based FC Difference 

**Figure 2:**
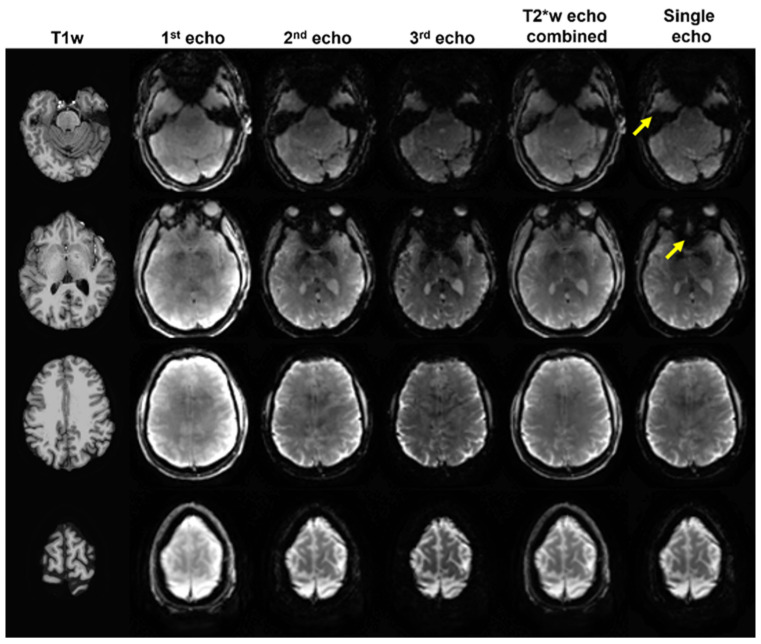
T1-weighted anatomical image and three echo time EPI images obtained on compact 3T. Echo combined EPI image shows signal intensity recovery in brain regions which suffers from signal dropout in single-echo EPI images as indicated. The yellow arrows indicate brain regions suffering from signal dropout with high magnetic susceptibility in EPI images.

**Figure 3:**
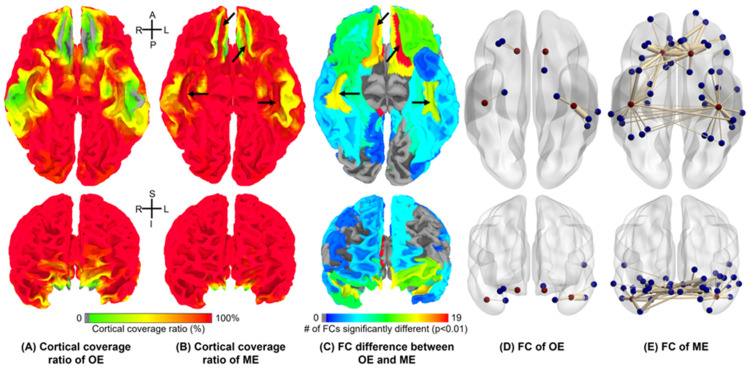
Cortical coverage ratio and functional connectivity. Cortical coverage ratio viewed on surface model. The cortical coverage was estimated by converting intensity-clipped images of (**A**) OE and (**B**) T_2_^*^-weighted echo-combined ME-EPI images onto a surface template. Cortical coverage ratio in frontal and temporal brain regions was dramatically improved with the multi-echo combination. (**C**) Functional connectivity between 168 ROIs defined in individual anatomical images were examined. Through the comparison between OE and ME group-level functional connectivity, the numbers of significantly different FCs on each ROI were evaluated. The indicated regions in (**B**,**C**) shows differences both in functional connectivity and cortical coverage ratio, which are left and right medial orbital sulci (olfactory sulci) and anterior transverse collateral sulci. The functional connections from the four regions are visualized by BrainNet Viewer with (**D**) OE and (**E**) ME datasets.

In Figure 2, raw EPI images with multiple echoes were plotted with the corresponding anatomical images of a single subject. The T_2_^*^-weighted echo combination enhanced signal intensity effectively in brain regions with high-susceptibility as indicated. For group comparison, the cortical coverage ratio was visualized in anterior and inferior views on the standard surface model for OE and ME datasets in Figure 3A,B, respectively. The overall cortical coverage ratio was improved with ME datasets. Specifically, medial orbital sulci (olfactory sulci) and anterior transverse collateral sulci on both hemispheres as indicated in Figure 3B showed signal intensity improvement across almost all subjects, which were medial orbital sulcus and anterior transverse collateral sulcus defined as ‘S_orbital_med-olfact’ and ‘S_collat_transv_ant’ in the reference [52], respectively.

Through the statistical comparison of 168-ROI-based FC matrices of OE and ME datasets, FC group difference of each ROI was visualized on the surface model as shown in Figure 3C. Four ROI regions, indicated by arrows in Figure 3C, had significant differences in functional connectivity, as well as in signal intensity. The locations of the four ROIs were consistent with the regions indicated in Figure 3B, where cortical coverage on the surface model was dramatically improved by echo combination. For visualization of the meaningful FCs from the four ROIs, i.e., |Fisher(r)| > 0.3, the BrainNet Viewer [62] was used to plot edges for inter-ROI functional connections derived from the four ROIs. In Figure 3D,E, OE datasets showed a much smaller number of FCs from the red nodes than ME datasets.

### 3.4. Seed-Based FC Extents

**Figure 4:**
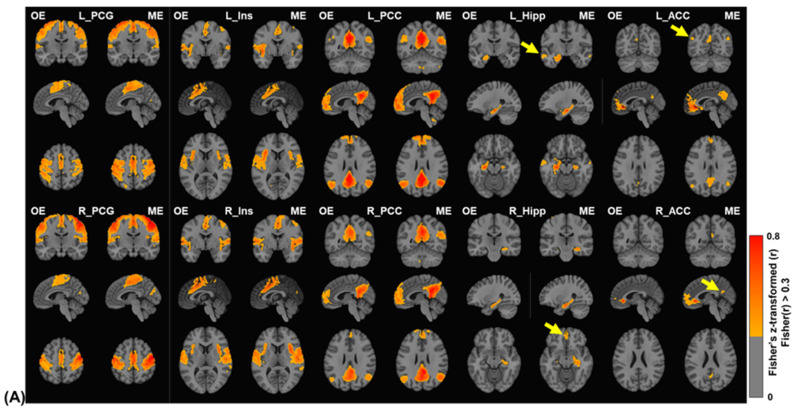

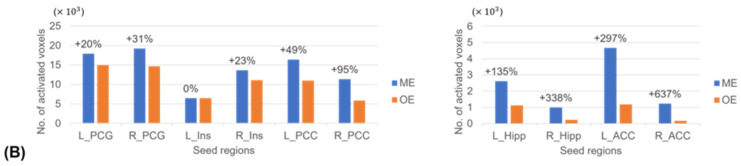
(**A**) Group-level seed-based functional connectivity of OE and ME datasets with 9-min-length datasets. Seed regions are precentral gyrus (PCG), insula (Ins), posterior cingulate cortex (PCC), hippocampus (Hipp), and anterior cingulate cortex (ACC) of left/right hemispheres (as a prefix of L_/R_) defined in AAL3v1 on MNI template. In visual inspection, the FC maps of ME group were compatible to those of OE group with seeds of PCG, Ins, and PCG and superior with seeds of Hipp and ACC to show additional FC clusters as indicated. The FC maps used in the analysis were thresholded with |Fisher(r)| > 0.3 after the statistical threshold of FDR-corrected *p* < 0.001. (**B**) Quantitative comparison of FC extents in the seed-based functional connectivity with 9-min-length datasets. The FC extents were calculated based on the group-level FC maps with statistical and functional thresholds. The number on each bar for ME-EPI shows the relative difference of FC extent between ME and OE. Note that Fisher(r) denoted Fisher-transformed Pearson correlation coefficient.

Seed-based group-level FC maps were obtained with datasets statistically thresholded with FDR-corrected *p* < 0.001. Each estimated FC extent was limited with |Fisher(r)| > 0.3. Note that Fisher(r) denoted Fisher-transformed Pearson correlation coefficient. Figure 4A showed average FC maps derived from 10 seeds as group results of 9-min-length OE and ME datasets. ME FC maps showed similar or stronger clusters than the OE FC maps for seeds placed in PCG, Ins, and PCC. For seeds placed in Hipp and ACC, ME datasets detected additional clusters in FC maps as indicated by arrows in Figure 4A. Figure 4B showed quantitative comparisons of the group-level FC extents with 9-min-length datasets, where ME produced broader FC extents with higher number of voxels than OE in most seed-based FC maps. Especially, for Hipp and ACC seeds, FC extents of ME datasets were approximately up to 3 times larger than those of OE datasets.

### 3.5. ICA-Based FC Detection

**Figure 5:**
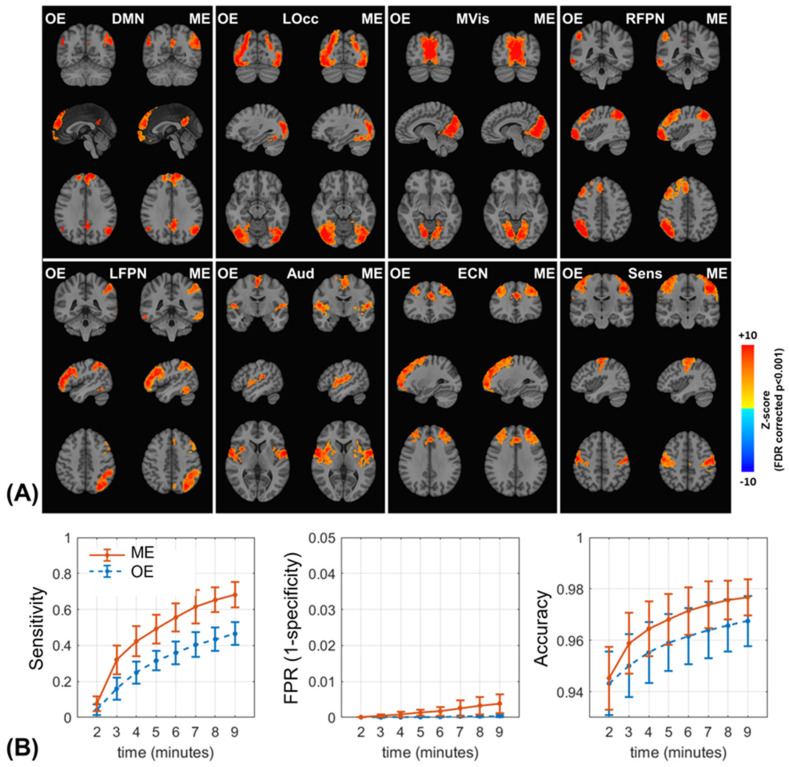
(**A**) Group-level ICA-derived brain networks of single- and multi-echo datasets with 9-min-length datasets. Dual regression and permutation testing (N = 5000) were performed to evaluate individual- and group-level IC maps, respectively. (**B**) Sensitivity, false positive rate, and accuracy were evaluated with single- and multi-echo datasets in ICA-derived brain networks. Eight IC maps obtained from Concat-ICA were assumed as ground truth for the representative brain networks. The single- and multi-echo group-level IC maps like (**A**) were calculated with a variety of scan times and were compared to the ground truth IC maps. Mean and standard deviation of the three metrics (columns) were evaluated across the eight brain networks on each scan time length. Abbreviations. DMN: default mode network, LOcc: lateral visual areas, MVis: medial visual areas, RFPN and LFPN: right and left frontal-parietal networks, respectively, Aud: auditory network, ECN: executive control network, and Sens: sensorimotor network.

Eight IC maps were identified as well-known intrinsic brain networks from 20 IC components. Group-level IC maps calculated by dual regression and permutation test with truncated OE/ME datasets are shown in Figure 5A. IC maps were derived by datasets thresholded by FDR corrected *p*-value < 0.001. Visually, group-level IC maps from ME datasets showed stronger and broader connectivity than those from OE datasets. Sensitivity, false positive rate (FPR, as ‘1-specificity’) and accuracy of OE/ME group IC maps were evaluated with a threshold of FDR corrected *p* < 0.001. In Figure 5B, the averages of all three metrics across eight IC maps were plotted against scan length. ME datasets produced higher sensitivity than OE datasets through all scan times. Scan lengths shorter than 2 min failed to show acceptable levels of sensitivity for both groups as expected. As sensitivity increased along scan length, although FPR increased for both groups, overall, it remained at a very low level (<0.004) for the threshold. The accuracy of the ME group was superior to that of the OE group at all scan time lengths. For example, a 4-min ME acquisition was comparable to an 8-min OE acquisition in terms of sensitivity and accuracy.

## 4. Discussion

In this study, the feasibility and capability of multi-echo fMRI was investigated on C3T with the high-performance gradient system. Three-echo fMRI was achieved with a spatial resolution of 2.4-mm isotropic voxel without partial Fourier sampling and a temporal resolution less than 1 s. The spatio-temporal resolution is comparable to the OE fMRI protocols used in landmark fMRI studies, such as HCP, ABCD and ADNI projects [7,8,9]. The shortened echo spacing enabled by higher slew rate allowed for additional echoes to be inserted at a relatively high spatial and temporal resolution with slight increase in TR. Since the ME acquisition extend the TR of OE from 700 ms to 940 ms, ME would yield fewer time frames than OE for a fixed amount of scan time. Our investigation strived to find out if the prospect of ME still holds despite of the loss of temporal resolution. The performance comparison of the ME over the OE acquisition with matched spatial resolution were assessed using signal variance and different types of functional connectivity analyses for further understanding. Compared to the OE acquisition, the ME acquisition was shown to have greatly reduced in thermal noise variance and recovered signal intensity/functional connectivity at brain regions suffering from high susceptibility. This resulted in a broad extent FC with several clusters in seed-based RSFC analysis, even in the regions of hippocampus and ACC suffering from distortions. Additionally, it was demonstrated that ME acquisition provided superior sensitivity and accuracy for detecting intrinsic brain networks in ICA-based RSFC analysis. Hence, a ME acquisition with higher TSNR and broader functional coverage was more beneficial than an OE acquisition with higher temporal resolution using the high-performance gradient on the C3T. With the advent of more high-performance gradient, such as the MAGNUS gradient insert [63] and Connectome 2.0 gradient [64], this study can shed some light to investigators who have access to these systems on which sequence to choose between ME-EPI or OE-EPI for RS-fMRI studies. 

**Figure 6:**
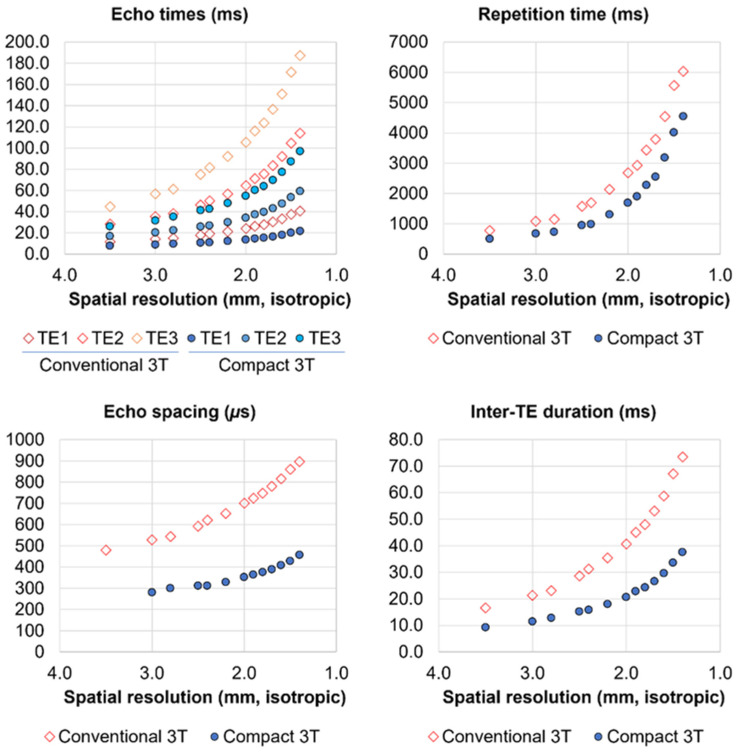
Potentials of the compact 3T scanner in multi-echo EPI imaging parameters (three echo times, repetition time, echo spacing and inter-TE duration) determined by isotropic voxel sizes. For a comparison, the imaging parameters available on conventional 3T scanner were given. In this study, spatial resolution was chosen to 2.4 mm with a repetition time less than 1 s.

The absence of a direct comparison with conventional whole-body 3T scanner is primarily due to limitations of whole-body scanner gradients. To achieve the same spatial resolution and brain coverage, the three echo times on whole-body scanner (i.e., GE 750 3T MRI scanner) would be 19.1 ms, 50.3 ms, and 81.5 ms, respectively. The last echo would be far out in the T_2_^*^ decay window and useless for signal contribution. As shown in Figure 6, an imaging parameter comparison at various isotropic spatial resolutions ranging from 1.4 mm to 3.5 mm showed a significant gap between the C3T and a conventional GE 750 scanner (200 T/m/s gradient slew rate and 50 mT/m peak gradient amplitude), where echo times, repetition time, echo spacing, and inter-TE duration were present depending on isotropic voxel size available on the C3T and conventional whole-body scanners. In this survey, full sampling in k-space was applied because partial Fourier sampling causes spatial resolution loss in phase-encoding direction. 2.4 mm imaging on the C3T scanner can achieve similar echo times, TR and inter-TE duration to those in 3.5 mm imaging on a conventional scanner. Even though the voxel volume in 2.4 mm imaging is only at 32.2% of the voxel volume in 3.5 mm imaging, it was demonstrated that 2.4 mm OE imaging could produce sufficient SNR for fMRI protocol in the functional MRI studies [7,8]. Thus, the study on the conventional whole-body 3T was omitted and we chose to only focus on the ME and OE comparison on the C3T. Additionally, our previous OE fMRI study between the C3T and the conventional 3T scanners demonstrated that the shortened echo spacing directly mitigates the geometric distortion on naïve EPI images to improve fidelity in a registration with anatomy images [35]. It was expected that this improvement is still valid for ME imaging on the C3T.

In this study, 3-echo acquisition was chosen based on the balanced consideration of T_2_^*^ decay window, temporal resolution, and number of echoes for the spatial resolution that we used as shown in Figure 6. The fourth echo would fall close to T_2_^*^~66.0 ms of a gray matter at 3T [65] and yield minimum signal, which would not contribute much to the combined-echo SNR. Additionally, any echoes added after the third one won’t be beneficial to signal recovery for a voxel with short T_2_^*^ because the first echo dominates the signal recovery in the T_2_^*^-weighted echo combination. On the other head, echoes with longer TE (>60 ms) could potentially be beneficial for voxels with long T_2_^*^ and helpful in accurate estimation of T_2_^*^ [3] or the automated classifier for TE-dependent signal, i.e., BOLD component in MEICA [54], which will be included in future study.

The signal variance in RS-fMRI could originate from various sources, such as pulsation and respiration, head motion, CSF fluctuation, hardware malfunction, and thermal noise, as well as BOLD signal. Preprocessing steps to improve sensitivity for functional connectivity have been developed to reduce the effect of artifacts caused by well-known sources, such as physiological fluctuation, head motion, and hardware instability [42,43,44,61]. For signal variance reduction related to thermal noise, smoothing and filtering have widely been used. For ME preprocessing, T_2_^*^-weighted echo combination has been used as a conventional method to boost contrast-to-noise and BOLD sensitivity [4]. We evaluated the impact of the echo combination on signal variance reduction. The reduced variance by echo combination was greater than from other regressors and tended to be uniformly distributed across the whole brain. Interestingly, the noise reduced by echo combination was not strongly correlated with other regressors and noise from adjacent voxels. This could be attributed to the fact that the variance removed by echo combination originated from thermal noise, since this is not strongly correlated with other artifact sources or the BOLD signal.

Along with the reduction in signal variance, ME fMRI produced the same or higher sensitivity for functional connectivity than the OE fMRI results via various FC analyses. This indicates that the small BOLD signal is well preserved during the echo combination and that the variance reduction is related to thermal noise rather than other noise/artifact sources. Since there is no specific regression model for thermal noise in the absence of temporal or spatial filtering, ME providing a higher SNR could be a preferred method over OE acquisition for RS-fMRI. Additionally, the *R*^2^ of the echo combination in WM appeared to be higher than in GM because WM has a lower level of signal variance than GM due to less BOLD signal variance and other physiological noise sources [66].

In the ICA-based FC evaluations, enhanced sensitivity was demonstrated for ME compared to OE acquisition. Although slightly higher FPRs were also observed for ME compared to OE acquisition, in all cases they are relatively low (<0.01) for all threshold values. In terms of accuracy, ME acquisition outperforms OE acquisition at statistical threshold with *p* < 0.001. The time dependency analysis indicated that ME acquisition with a shorter scan time could be considered as a better substitute to longer OE acquisition for RS-fMRI studies as they showed comparable sensitivity and accuracy.

Previous task-based BOLD fMRI studies have shown that signal intensity enhancement by echo combination offers the opportunity to study regions with rapid susceptibility spatial variation, especially along the inferior cerebral hemispheres, such as the ventromedial prefrontal cortex [39,67]. Inter-ROI FC comparison in this study with high performance gradients supports that signal intensity enhancement by echo combination helps to extract even spontaneous BOLD signal in regions of high susceptibility, such as medial orbital sulci (olfactory sulci) and anterior transverse collateral sulci. Echo combination has yet to fully recover signal dropout that intrinsically occurs in EPI acquisition. When it is combined with other susceptibility reduction techniques, such as z-shimming [68], it is expected to further improve cortical coverage.

ICA-based fMRI de-noising methods, such as ICA-AROMA, MEICA, TEDANA, etc. [54,69] has shown the potential for data quality improvements, as well as the capability to subject-specifically separate signal versus noise/artifacts. These methods could provide noise and artifact component removal and, thus, improved performance for FC detection [25,70]. The primary focus of this work is to compare the benefit of only ME combination to the shorter (but comparable) TR of OE acquisition on the condition of high-spatial-temporal resolution implementation, especially when it is performed on the C3T fully using the high gradient slew rate. However, the IC classifiers for these ICA-based de-noising methods were extensively studied with relatively higher SNR fMRI data, such as low-spatial-temporal resolution fMRI datasets. As described in previous studies [22,69,71], the classifiers could fail in case of ambiguous components and an expert/interpreter may be required to manually intervene and decide whether components were classified correctly. For these reasons, ICA-based de-noising methods were excluded in preprocessing of data cleaning in this study, which would be examined in future study. Instead of detecting ICA-based subject-specific artifact components, concat-ICA was applied to detect ‘well-known’ brain network components common to all subjects and all datasets. Echo combination served as a single extra pre-processing step for the ME fMRI. All the other denoising steps remained the same between ME and OE acquisitions. Even without ICA-based denoising pre-processing, ME fMRI still outperforms OE fMRI based on several analysis methods. 

A limitation in this study was that the scan length of the ME acquisition was restricted by a scanner software limit on the total number of reconstructed images per scan. With the limit, the proposed ME imaging protocol (Table 1) was restricted to only 5 min per scan. To achieve a longer scan length, two ME scans had to be performed consecutively and concatenated together afterwards. Due to this, there is a potential loss in temporal continuity introduced by data truncation and concatenation. Since RS FC estimates are modulated by slow frequency dynamics with cycles on the order of several minutes [72], data truncation of any RS-fMRI dataset could cause biased reliability and reproducibility in FC estimate [73]. As an alternative approach, data scrubbing, such as delta function regression has been suggested to minimize a loss in reliability and reproducibility on the FC estimate [73]. Although the previous study demonstrated that data concatenation could improve the reliability of functional connectomics [48], it is still unclear how a potential loss in temporal continuity caused by the data concatenation would influence the FC estimate [48]. With a recent software upgrade of the C3T scanner, the system currently allows a non-stop acquisition of the ME scan over 10 min. This enables comparison of concatenated data with data acquired without discontinuity. As part of future works, the effect of data concatenation with different preprocessing steps will be investigated. 

For future works, the enhanced sensitivity of ME fMRI will be applied to clinical RS-fMRI studies of aging and neurodegenerative diseases, such as dementia as the brain suffers from signal dropout in regions with iron deposition and increased partial volume effect due to brain atrophy [74]. Additionally, ME fMRI provides opportunities for improved functional connectivity detection in regions, such as the hippocampus and ACC, which would benefit cognitive neuroscience studies related to memory or cognition [75]. From a technical point of view, the choice of echo number for ME fMRI needs to be evaluated when ICA-based fMRI methods are used for denoising and motion correction. The ME acquisition studied here is based on 2D EPI acquisition. Emerging new techniques, such as wave-CAIPI [76,77] could potentially enable 3D ME imaging. The high-performance gradient on the C3T would also be beneficial for shortening the sinusoidal acquisition window and facilitate ME fMRI, which makes it an interesting topic for future study. 

## 5. Conclusions

Multi-echo-EPI acquisition was feasible with a spatial resolution < 2.5 mm isotropic voxel and a temporal resolution < 1 s on a high-performance gradient system of C3T and was demonstrated to provide superior SNR, TSNRs, sensitivity and accuracy to the corresponding OE-EPI acquisition with matching spatial resolution despite the temporal resolution loss. The high-performance gradient enabled high-spatial-temporal resolution ME EPI would be the method of choice for RS-fMRI study on the C3T.

## Figures and Tables

**Table 1:** Details of imaging parameters for single- and multi-echo EPI acquisitions.

Imaging Parameter	Single-Echo EPI	Multi-Echo EPI
Field of view (FOV, mm)	224 × 224	
Matrix size	94 × 94	
In-plane resolution (mm)	2.38 × 2.38	
k-space partial Fourier factor	No partial Fourier	
Slice thickness (mm)	2.4	
Number of slices	52	
Inter-slice gap (mm)	0.3	
Inferior-to-superior coverage (mm)	140.4	
In-plane acceleration factor	2	
Multi-band factor	4	
Echo spacing (µs)	352	
Receiver bandwidth (Hz)	±250 k	
Echo time (TE, ms)	30	11.9, 29.8, 47.8
Repetition time (TR, ms)	700	940
RF flip angle (FA, °) ^1^	52	59
Number of volumes per scan	961	320
Scan duration (mm:ss)	11:12	5:00
Note	-	Scan twice

^1^ FAs were applied based on the calculated Ernst angles assuming T_1_ of 1.4 s in gray matter at 3T.

## Data Availability

The original contributions presented in the study are included in the article, further inquiries can be directed to the corresponding author.
